# COVID-19 findings identified in chest computed tomography: a pictorial essay

**DOI:** 10.31744/einstein_journal/2020RW5741

**Published:** 2020-06-18

**Authors:** Marcela Emer Egypto Rosa, Marina Justi Rosa de Matos, Renata Silveira Olimpio de Paula Furtado, Vanessa Mizubuti Brito, Lucas Tadashi Wada Amaral, Gabriel Laverdi Beraldo, Eduardo Kaiser Ururahy Nunes Fonseca, Rodrigo Caruso Chate, Rodrigo Bastos Duarte Passos, Gustavo Borges da Silva Teles, Murilo Marques Almeida Silva, Patrícia Yokoo, Elaine Yanata, Hamilton Shoji, Gilberto Szarf, Marcelo Buarque de Gusmão Funari

**Affiliations:** 1 Hospital Israelita Albert Einstein São PauloSP Brazil Hospital Israelita Albert Einstein, São Paulo, SP, Brazil.

**Keywords:** Coronavirus infections, Coronavirus, COVID-19, Multidetector computed tomography

## Abstract

The disease caused by the new coronavirus, or COVID-19, has been recently described and became a health issue worldwide. Its diagnosis of certainty is given by polymerase chain reaction. High-resolution computed tomography, however, is useful in the current context of pandemic, especially for the most severe cases, in assessing disease extent, possible differential diagnoses and searching complications. In patients with suspected clinical symptoms and typical imaging findings, in which there is still no laboratory test result, or polymerase chain reaction is not available, the role of this test is still discussed. In addition, it is important to note that part of the patients present false-negative laboratory tests, especially in initial cases, which can delay isolation, favoring the spread of the disease. Thus, knowledge about the COVID-19 and its imaging manifestations is extremely relevant for all physicians involved in the patient care, clinicians or radiologists.

## INTRODUCTION

Coronavirus disease (COVID-19), caused by the severe acute respiratory syndrome coronavirus 2 (SARS-CoV-2), was first described in December 2019, in the city of Wuhan, in the province of Hubei, in China. Easily transmitted among humans, the disease quickly became a worldwide health concern.^( 1 , 2 )^

Its diagnosis is confirmed by reverse transcriptase polymerase chain reaction (RT-PCR). However, as recently demonstrated in Italy, laboratories can quickly become overwhelmed with results delay and lack of kits, hindering diagnosis of patients and early isolation, and thus favoring an increase in virus transmission.^( 3 )^ Some studies have demonstrated an initial non-negligible false-negative rate, even in symptomatic patients - part of them already presenting characteristic imaging abnormalities in chest computed tomography (CT) and only later becoming positive on laboratory results.^( 4 , 5 )^ Although CT is not indicated as the only diagnostic test by several medical specialty societies,^( 6 , 7 )^ it is a valuable diagnostic tool for these patients, and it is also useful to monitor progression of disease and to detect possible complications. It is worth mentioning that imaging findings do not replace RT-PCR for diagnosis.^( 2 , 6 )^

The usual chest CT protocol is performed with 1.0mm slice thickness, and if possible, low-dose without intravenous contrast.^( 6 )^

There may be overlapping findings of some tomographic alterations related to COVID-19 with the ones found in other viral infections. Although not pathognomonic, some of those findings have characteristics that stand out suggesting the disease.^( 8 )^ The most characteristic finding consist of multiple ground-glass opacities, sometimes rounded, mostly in the periphery of pulmonary lobes and in posterior regions, often in the bases. The involvement is mostly bilateral and multilobar, and may evolve to crazy paving pattern and coalescent consolidations.

Airway involvement, lymph node enlargement, excavations, lobar consolidations, nodules or predominance of perihilar changes are not common. When these findings are present, co-infection or even other diagnoses should be considered.^( 9 - 13 )^

The objective of this article was to present examples of tomographic findings described in pneumonia caused by COVID-19, so that healthcare professionals working during this pandemic can be familiar with the disease and identify suspected patients quicker.

The project was approved by the Research Ethics Committee of *Hospital Israelita Albert Einstein* , CAAE: 30634120.1.0000.0071 and oficial opinion 4.086.306.

## GROUND-GLASS OPACITY

Ground-glass opacities are defined as slight increase in pulmonary density, without obscuring vessel walls and bronchi. The cause may be partial filling of air spaces and/or interstitial thickening, which are found in processes of diverse etiologies, infectious (by different agents) or not.^( 14 , 15 )^

**Figure 1:**
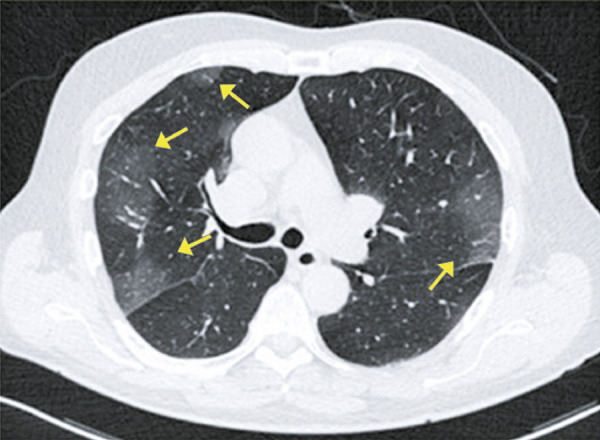
A 75-year-old patient with COVID-19, presenting respiratory discomfort for 3 days, and fever for one day. Computed tomography showing peripheral ground-glass opacities in the upper lobes

**Figure 2:**
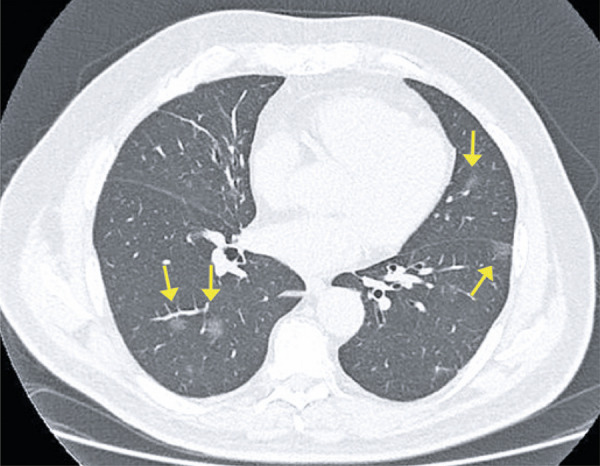
A 72-year-old patient with COVID-19, presenting cough, fever and dyspnea for four days. Computed tomography image show multifocal rounded ground-glass opacities, with typical aspect and distribution of COVID-19

Ground-ground-glass opacities are the most common and early finding (approximately zero to 4 days after onset of symptoms) in COVID-19 patients; they often present bilateral, peripheral and subpleural distribution in the lower lobes ( Figures 1 and 2 ).

## CRAZY PAVING

**Figure 3:**
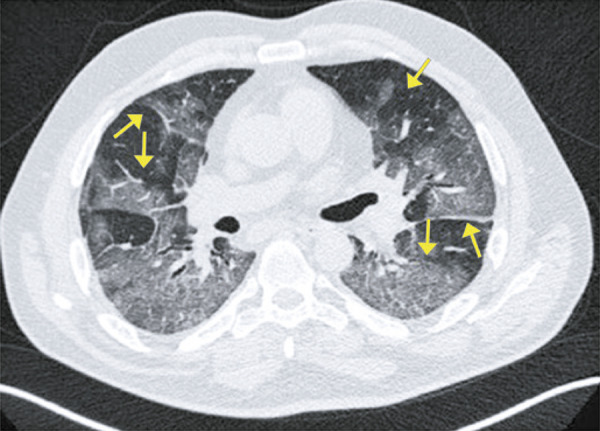
A 63-year-old patient with COVID-19, presenting dyspnea, fever, chills and myalgia for four days. Computed tomography showing ground-glass opacities associated with reticulations (crazy paving pattern)

**Figure 4:**
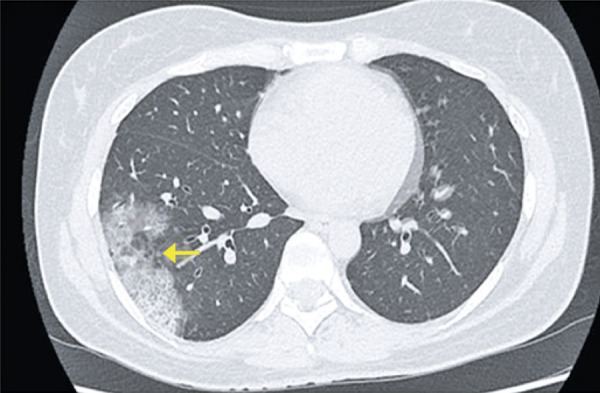
A 44-year-old patient with COVID-19, presenting headache, odinophagia, productive cough, dyspnea and myalgia for six days. Computed tomography showing ground-glass opacity in the periphery of the right lower lobe, associated with reticulations and interlobular septum thickening (crazy paving pattern)

**Figure 5:**
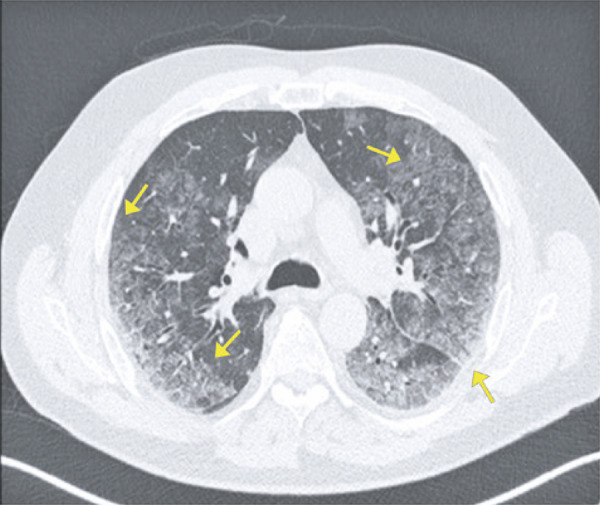
A 38-year-old patient with COVID-19, presenting fever and dry cough for six days. Computed tomography showing diffuse ground-glass opacities associated with thickening of interlobular septa and reticulations (crazy paving)

Ground-ground-glass opacities are sometimes superimposed with septal thickening located inside or amidst the secondary pulmonary lobules; that is, intralobular and interlobular septa. These superimposed findings are called crazy paving pattern.^( 14 , 15 )^ Ground glass opacities are not specific for viral infection, and can be found in several viral diseases, or even in non-infectious diseases. It is frequently present in acute respiratory distress syndrome (ARDS), indicating heterogenous alveolar damage, due to severe pneumonia. In the context of COVID-19, it is more often characterized some days after onset of symptoms ( Figures 3 to 5 ).

## PLEURAL EFFUSION

**Figure 6:**
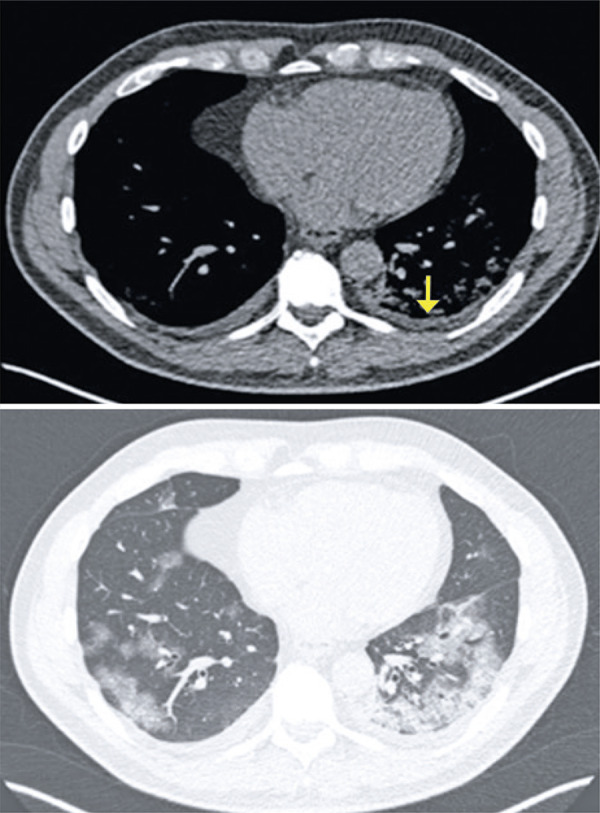
A 63-year-old patient with COVID-19, presenting fever and myalgia seven days. Computed tomography shows bilateral small-volume pleural effusion, ground-glass opacities in the lower lobes and consolidation in the left lower lobe

Pleural effusion is more frequent in patients with more severe disease and may suggest a poorer prognosis^( 10 , 11 )^ ( Figure 6 ).

## REVERSED HALO SIGN

**Figure 7:**
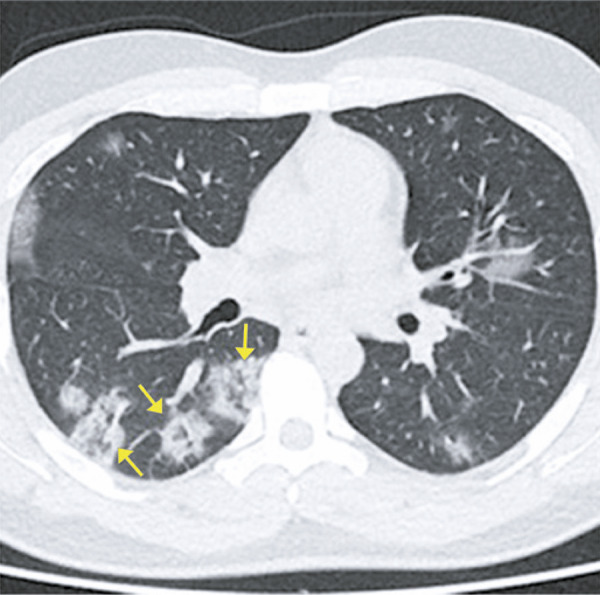
A 38-year-old patient with COVID-19, presenting fever, dry cough, malaise and headache for six days. Computed tomography demonstrating reversed halo sign in the lower lobes

**Figure 8:**
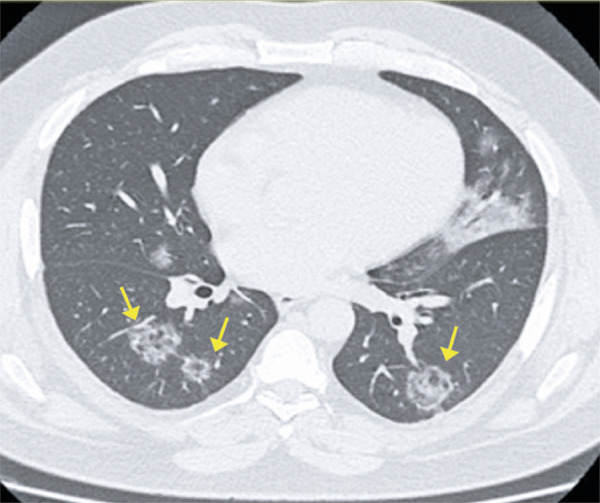
A 43-year-old patient with COVID-19, presenting shortness of breath, fever and cough for eight days. Computed tomography showing reversed halo sign in the lower lobes

The reversed halo sign is described as a central area of ground-glass opacity, surrounded by a more or less complete ring of consolidation.^( 14 , 15 )^ It was originally described as a specific finding for cryptogenic organizing pneumonia; however, it was later observed in patients with several other diseases, such as COVID-19^( 11 )^ ( Figures 7 and 8 ).

## CONSOLIDATION

**Figure 9:**
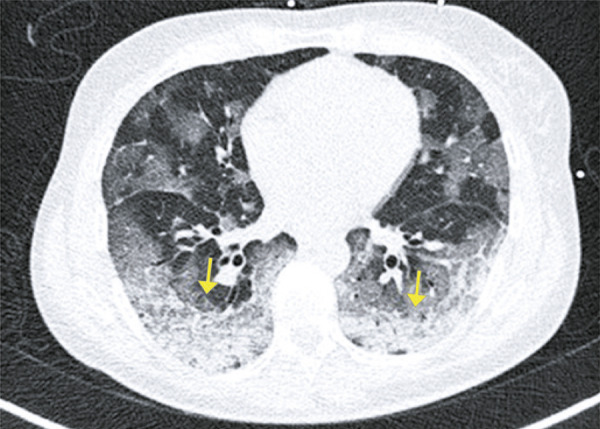
A 51-year-old patient presenting with dry cough and headache for seven days, and fever, for 5 days. Computed tomography showing diffuse ground-glass opacities, associated with peripheral consolidations in the lower pulmonary lobes

**Figure 10:**
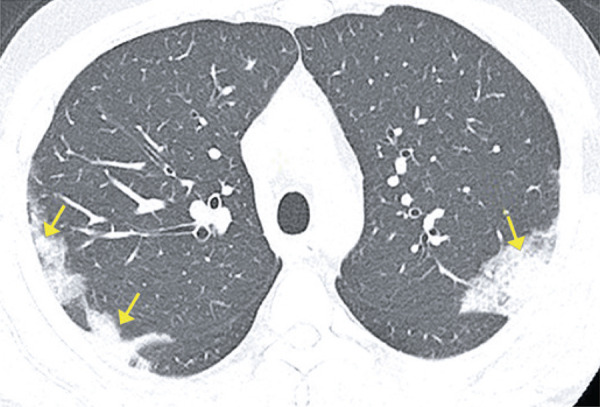
A 41-year-old patient with COVID-19, presenting cough, fever and dyspnea for five days. Computed tomography showing peripheral consolidations in the upper lobes

**Figure 11:**
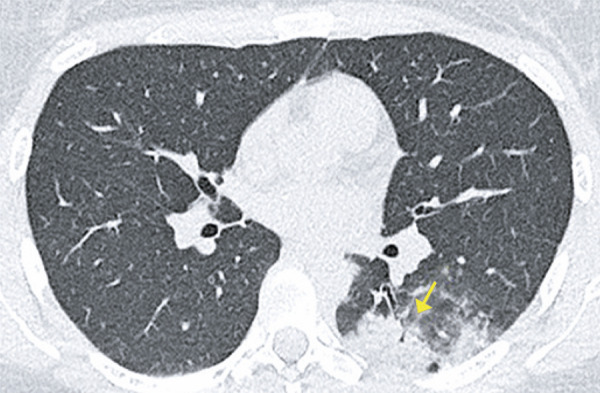
A 28-year-old patient with COVID-19, presenting cough for 15 days. Computed tomography showing peripheral consolidation in the left lower lobe

**Figure 12:**
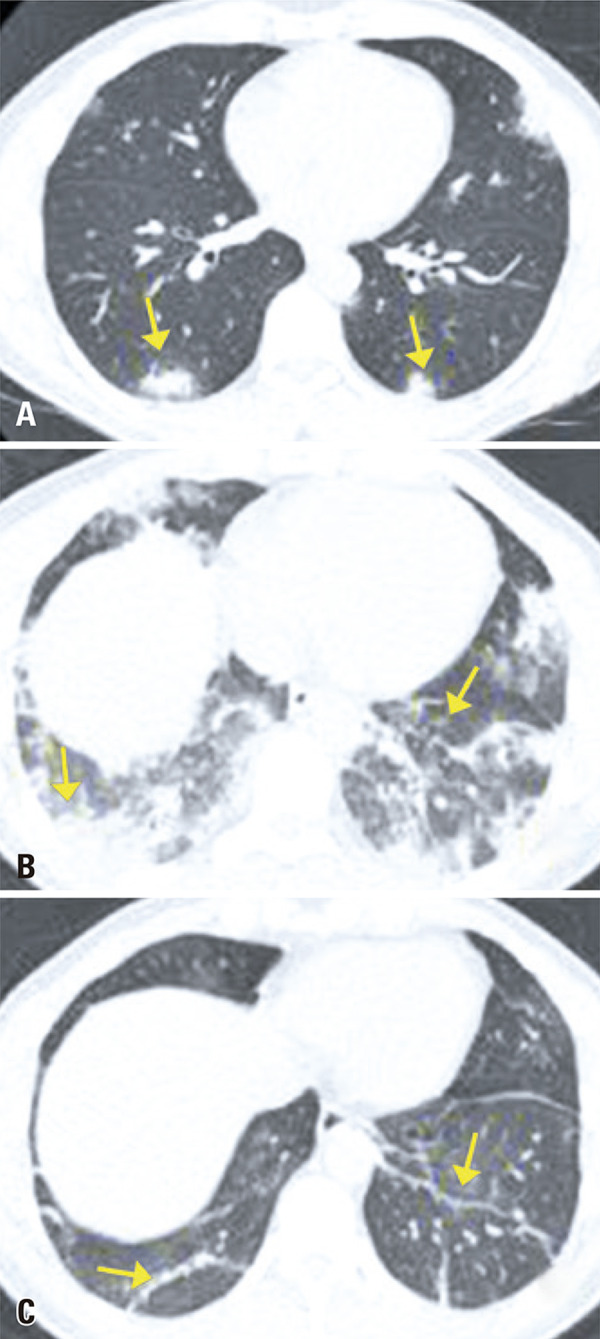
Computed tomography images of a 44-year-old patient with COVID-19. (A) Exam performed on the first day of disease, showing peripheral consolidations; (B) Exam during the fifth day of disease, showing significant increase in extent and density of pulmonary opacities, predominating consolidation; (C) Exam on the tenth day of disease, showing decreased disease extent and reduced density of the previously observed pulmonary abnormalities, persisting sparse pulmonary parenchymal bands in the periphery of lower fields - finding usually described in the late phase of the disease

Consolidation is the second most frequent change found in pneumonia caused by COVID-19, after ground-glass opacities, and tends to occur in the later phases of infection, mainly after the tenth day.^( 10 - 12 )^ Very often, both changes are observed together. It represents filling of the alveoli by inflammatory exsudate. This radiological finding is characterized by increased pulmonary density with obscured vessels and interstitial lines, and often present a round shape in this viral pneumonia^( 14 , 15 )^ ( Figures 9 to 11 ). The progressive pattern of ground glass, crazy paving and consolidations is shown in figure 12 , which also demonstrate residual parenchymal bands in the last exam; such findings have been described in the late phase of convalescence of these patients.^( 11 )^

## AIR BRONCHOGRAM

**Figure 13:**
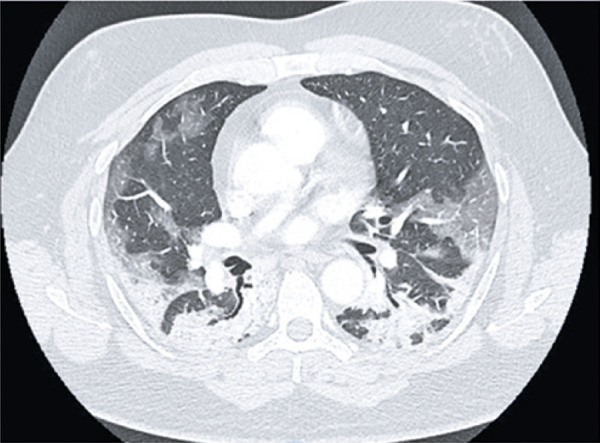
A 51-year-old patient with COVID-19, presenting fever and cough for five days. Computed tomography showing peripheral ground-glass opacities, posterior consolidations with air bronchograms

Air bronchogram is the identification of air-filled bronchi, inside an alveolar consolidation.^( 14 , 15 )^ Radiologically, it is characterized as a hypoattenuating tubular structure amidst a consolidated pulmonary parenchyma ( Figure 13 ).

## LYMPHADENOPATHY

**Figure 14:**
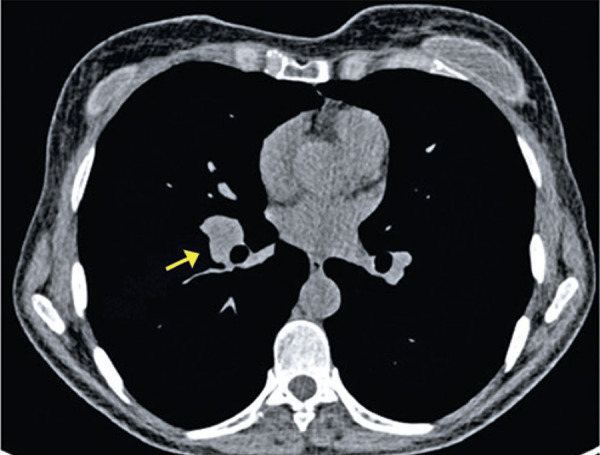
A 52-year-old patient with COVID-19, presenting dyspnea, fever, and myalgia for three days. Computed tomography shows hilar lymphadenopathy on the right (arrow)

Thoracic lymphadenopathy is characterized by mediastinal and/or hilar lymph nodes, measuring over than 1cm in its smallest axial axis. Computed tomography just provides the dimensions and morphology of these lymph nodes, and it is not possible to differentiate them from primary and/or secondary neoplastic disease. Enlarged lymph nodes are not common in COVID-19^( 14 , 15 )^ ( Figure 14 ).

## CONCLUSION

Although the diagnosis of COVID-19 can only be confirmed by polymerase chain reaction, computed tomography can assist in assessing the extent of the disease, possible complications and establishing alternative diagnoses. It is important that the medical team to be familiar with the imaging findings suggestive of viral pneumonia compatible with COVID-19.

## References

[1] 1. Pan F, Ye T, Sun P, Gui S, Liang B, Li L, et al. Time course of lung changes at chest CT during recovery from coronavirus disease 2019 (COVID-19). Radiology. 2020;295(3):715-21. 10.1148/radiol.2020200370 PMC7233367 32053470

[2] 2. Zu ZY, Jiang MD, Xu PP, Chen W, Ni QQ, Lu GM, et al. Coronavirus Disease 2019 (COVID-19): a perspective from China. Radiology. 2020;200490. doi: 10.1148/radiol.2020200490. [Epub ahead of print]. 10.1148/radiol.2020200490 PMC7233368 32083985

[3] 3. Grasselli G, Pesenti A, Cecconi M. Critical care utilization for the COVID-19 outbreak in Lombardy, Italy: early experience and forecast during an emergency response. JAMA. 2020. doi: 10.1001/jama.2020.4031. [Epub ahead of print]. 10.1001/jama.2020.4031 32167538

[4] 4. Xie X, Zhong Z, Zhao W, Zheng C, Wang F, Liu J. Chest CT for typical 2019-nCoV pneumonia: relationship to negative RT-PCR testing. Radiology. 2020;200343. doi: 10.1148/radiol.2020200343. [Epub ahead of print]. 10.1148/radiol.2020200343 PMC7233363 32049601

[5] 5. Huang P, Liu T, Huang L, Liu H, Lei M, Xu W, et al. Use of chest CT in combination with negative RT-PCR assay for the 2019 novel coronavirus but high clinical suspicion. Radiology. 2020;295(1):22-3. 10.1148/radiol.2020200330 PMC7233360 32049600

[6] 6. Colégio Brasileiro de Radiologia e Diagnóstico por Imagem (CBR). Recomendações de uso de métodos de imagem para pacientes suspeitos de infecção pelo COVID-19 [Internet]. São Paulo: CBR; 2020 [citado 2020 Abr 16]. Disponível em: https://cbr.org.br/wp-content/uploads/2020/03/CBR_Recomenda%C3%A7%C3%B5es-de-uso-de-m%C3%A9todos-de-imagem.pdf

[7] 7. American College of Radiology (ACR). ACR Recommendations for the use of Chest Radiography and Computed Tomography (CT) for Suspected COVID-19 Infection [Internet]. ACR; 2020 [cited 2020 Mar 16]. Available from: https://www.acr.org/Advocacy-and-Economics/ACR-Position-Statements/Recommendations-for-Chest-Radiography-and-CT-for-Suspected-COVID19-Infection

[8] 8. Hosseiny M, Kooraki S, Gholamrezanezhad A, Reddy S, Myers L. Radiology perspective of coronavirus disease 2019 (COVID-19): lessons from severe acute respiratory syndrome and middle east respiratory syndrome. AJR Am J Roentgenol. 2020;214(5):1078-82. 10.2214/AJR.20.22969 32108495

[9] 9. Ye Z, Zhang Y, Wang Y, Huang Z, Song B. Chest CT manifestations of new coronavirus disease 2019 (COVID-19): a pictorial review. Eur Radiol. 2020. 10.1007/s00330-020-06801-0. [Epub ahead of print]. Review. PMC7088323 32193638

[10] 10. Chate RC, Fonseca EK, Passos RB, Teles GB, Shoji H, Szarf G. Presentation of pulmonary infection on CT in COVID-19: initial experience in Brazil. J Bras Pneumol. 2020;46(2):e20200121. 10.36416/1806-3756/e20200121 PMC7462704 32294718

[11] 11. Bernheim A, Mei X, Huang M, Yang Y, Fayad ZA, Zhang N, et al. Chest CT findings in coronavirus disease-19 (COVID-19): relationship to duration of infection. Radiology. 2020;295(3):200463. 10.1148/radiol.2020200463 PMC7233369 32077789

[12] 12. Wang Y, Dong C, Hu Y, Li C, Ren Q, Zhang X, et al. Temporal changes of CT findings in 90 patients with COVID-19 pneumonia: a longitudinal study. Radiology. 2020;200843. doi: 10.1148/radiol.2020200843. [Epub ahead of print]. 10.1148/radiol.2020200843 PMC7233482 32191587

[13] 13. Simpson S, Kay FU, Abbara S, Bhalla S, Chung JH, Chung M, et al. Radiological Society of North America Expert Consensus Statement on Reporting Chest CT Findings Related to COVID-19. Endorsed by the Society of Thoracic Radiology, the American College of Radiology, and RSNA. Radiol Cardiothorac Imaging. 2020;2(2). 10.1148/ryct.2020200152 PMC7233447 33778571

[14] 14. Hansell DM, Bankier AA, MacMahon H, McLoud TC, Muller NL, Remy J. Fleischner society: glossary of terms for thoracic imaging. Radiology. 2008;246(3):697-722. 10.1148/radiol.2462070712 18195376

[15] 15. Silva CI, Marchiori E, Souza Júnior AS, Müller NL. Consenso brasileiro ilustrado sobre a terminologia dos descritores e padrões fundamentais da TC de tórax. J Bras Pneumol. 2010;36(1):99-123. 10.1590/s1806-37132010000100016 20209314

