# Supplementary material for: COVID-19 findings identified in chest computed tomography: a pictorial essay
Source: Einstein (Sao Paulo). 2020 Jun 18;18:eRW5741. doi: 10.31744/einstein_journal/2020RW5741 (PMC7342047; doi:10.31744/einstein_journal/2020RW5741)
Supplement: Translation

# Translation: Achados da COVID-19 identificados na tomografia computadorizada de tórax: ensaio pictórico

**Authors:** Marcela Emer Egypto Rosa, Marina Justi Rosa de Matos, Renata Silveira Olimpio de Paula Furtado, Vanessa Mizubuti Brito, Lucas Tadashi Wada Amaral, Gabriel Laverdi Beraldo, Eduardo Kaiser Ururahy Nunes Fonseca, Rodrigo Caruso Chate, Rodrigo Bastos Duarte Passos, Gustavo Borges da Silva Teles, Murilo Marques Almeida Silva, Patrícia Yokoo, Elaine Yanata, Hamilton Shoji, Gilberto Szarf, Marcelo Buarque de Gusmão Funari

**Affiliations:** 1 Brasil Hospital Israelita Albert Einstein, São Paulo, SP, Brasil.

**Keywords:** Infecções por coronavírus, Coronavírus, COVID-19, Tomografia computadorizada multidetectores

## Abstract

A doença causada pelo novo coronavírus, ou COVID-19, foi descrita recentemente e tornou-se uma questão de saúde mundial. Seu diagnóstico de certeza é dado pela reação em cadeia da polimerase. A tomografia computadorizada de alta resolução, entretanto, mostra-se útil no contexto atual de pandemia, especialmente nos casos mais graves, na avaliação da extensão da doença, em possíveis diagnósticos diferenciais e na pesquisa de complicações. Em pacientes com quadro clínico suspeito e achados de imagem típicos, nos quais ainda não há resultado laboratorial ou a reação em cadeia da polimerase não se encontra disponível, ainda se discute o papel desse exame. Importante ressaltar que parte dos pacientes apresenta exames laboratoriais falsos-negativos, notadamente em casos iniciais, o que pode retardar medidas de isolamento, favorecendo a propagação da doença. Dessa forma, o conhecimento da COVID-19 e de suas manifestações nos exames de imagem é de extrema importância para os médicos envolvidos no atendimento, sejam clínicos ou radiologistas.

## INTRODUÇÃO

A doença pelo coronavírus 2019 (COVID-19), causada pelo novo coronavírus, denominado SARS-CoV-2, foi descrita, pela primeira vez, em dezembro de 2019 na cidade de Wuhan, na província de Hubei, na China. Com fácil transmissão entre humanos, a doença rapidamente tornou-se questão de saúde mundial.^( 1 , 2 )^

Sua confirmação diagnóstica se dá por meio da reação em cadeia da polimerase por transcrição reversa (RT-PCR), porém, como foi demonstrado em recente experiência da Itália, pode haver uma rápida saturação dos laboratórios, com demora crescente para a liberação de resultados e esgotamento dos *kits* , não sendo possível realizar o diagnóstico e o isolamento precoce de parte dos pacientes, favorecendo o aumento da transmissão.^( 3 )^Alguns estudos demonstraram taxa não desprezível inicial de falsos-negativos, mesmo em pacientes sintomáticos – parte dos quais já demonstravam alterações clássicas e características na tomografia computadorizada (TC) de tórax e que somente posteriormente se tornaram positivos laboratorialmente para a doença.^( 4 , 5 )^A TC, muito embora não seja indicada como exame de escolha isolado para o diagnóstico pelas diversas sociedades de especialidade,^( 6 , 7 )^torna-se valiosa ferramenta de auxílio diagnóstico nesses pacientes, além de ser útil no acompanhamento da evolução e na detecção de possíveis complicações. Importante salientar que os achados de imagem não substituem a RT-PCR para o diagnóstico.^( 2 , 6 )^

O protocolo usual é o de tomografia de tórax com espessura de corte de 1,0mm, se possível em protocolo de baixa dose de radiação e sem contraste endovenoso.^( 6 )^

Ainda que não patognomônicas e que possam se sobrepor aos achados de outras infecções virais, algumas alterações tomográficas da COVID-19 têm características que as destacam daquelas vistas em outras infecções, muitas vezes sendo bastante sugestivas desta infecção em particular.^( 8 )^O achado mais característico é o de múltiplas opacidades em vidro fosco, por vezes de morfologia arredondada, predominando na periferia dos lobos pulmonares e em suas regiões posteriores, frequentemente nas bases. O acometimento é, na maioria das vezes, bilateral e multilobar, podendo evoluir para padrão de pavimentação em mosaico ( *crazy paving* ) e coalescer em consolidações com a evolução da infecção.

Não são comuns o acometimento das vias aéreas, as linfonodomegalias, as escavações, as consolidações lobares, os nódulos ou mesmo o predomínio peri-hilar das alterações. Quando esses achados estão presentes, superposição de infecções ou, ainda, um diagnóstico alternativo devem ser aventados.^( 9 - 13 )^

O objetivo deste artigo foi apresentar exemplos dos achados tomográficos descritos na pneumonia por COVID-19 para que todos que atuam nessa pandemia fiquem familiarizados e possam identificar mais rapidamente pacientes com suspeita para essa afecção.

O trabalho foi aprovado pelo Comitê de Ética em Pesquisa do Hospital Israelita Albert Einstein com CAAE: 30634120.1.0000.0071 e número do parecer 4.086.306.

## OPACIDADE EM VIDRO FOSCO

Opacidades em vidro fosco são definidas como discreto aumento da densidade pulmonar, sem obscurecimento dos vasos e brônquios. Pode ter como causa o preenchimento parcial dos espaços aéreos e/ou espessamento do interstício, sendo encontrada em processos de diversas etiologias, infecciosas (por diversos agentes) e não infecciosas.^( 14 , 15 )^

As opacidades em vidro fosco são o achado mais comum e mais precoce (cerca de zero a 4 dias após o início dos sintomas) nos pacientes com COVID-19, sendo encontradas mais comumente com distribuição bilateral, periférica e subpleural nos lobos inferiores ( Figuras 1 e 2 ).

**Figura 1:** Paciente de 75 anos com COVID-19, apresentando desconforto respiratório, há 3 dias, e febre, há 1 dia. Tomografia computadorizada mostrando opacidades em vidro fosco periféricas nos lobos superiores

**Figura 2:** Paciente de 72 anos com COVID-19, apresentando tosse, febre e dispneia, há 4 dias. As imagens da tomografia demonstram opacidades arredondadas em vidro fosco multifocais, de aspecto e distribuição típicos da COVID-19

## PAVIMENTAÇÃO EM MOSAICO

As opacidades em vidro fosco, por vezes, são sobrepostas ao espessamento dos septos localizados dentro e entre os lóbulos pulmonares secundários – respectivamente os septos intra e interlobulares. Tais achados sobrepostos são denominados de pavimentação em mosaico, ou *crazy paving* .^( 14 , 15 )^O vidro fosco não é específico de uma infecção viral, podendo ser encontrado em diversas delas e também ter origem não infecciosa. No contexto da COVID-19, é mais comumente caracterizado após alguns dias do início dos sintomas ( Figuras 3 a 5 ).

**Figura 3:** Paciente de 63 anos com COVID-19, apresentando dispneia, febre, calafrios e mialgia há 4 dias. Tomografia computadorizada mostrando opacidades em vidro fosco associadas a reticulações (pavimentação em mosaico)

**Figura 4:** Paciente de 44 anos com COVID-19 apresentando cefaleia, odinofagia, tosse secretiva, dispneia e mialgia há 6 dias. Tomografia computadorizada mostrando opacidade em vidro fosco periférica no lobo inferior direito, associada a reticulações e espessamento dos septos interlobulares (padrão de pavimentação em mosaico)

**Figura 5:** Paciente de 38 anos com COVID-19, apresentando febre e tosse seca há 6 dias. Tomografia computadorizada mostrando opacidades em vidro fosco difusas associadas a espessamento dos septos interlobulares e reticulações (pavimentação em mosaico)

## DERRAME PLEURAL

A presença de derrame pleural tem maior incidência em pacientes mais graves e pode sugerir um pior prognóstico^( 10 , 11 )^( Figura 6 ).

**Figura 6:** Paciente de 63 anos com COVID-19, apresentando febre e mialgia há 7 dias. Tomografia computadorizada demonstrando derrame pleural bilateral de pequeno volume e opacidades em vidro fosco nos lobos inferiores, além de consolidação no lobo inferior esquerdo

## SINAL DO HALO INVERTIDO

O sinal do halo invertido é descrito como área focal arredondada de opacidade em vidro fosco, cercada por uma área anelar de consolidação.^( 14 , 15 )^Originalmente foi descrito como achado específico para pneumonia em organização criptogênica, porém, posteriormente, foi observado também em pacientes com diversas outras doenças, como nos casos de COVID-19^( 11 )^( Figuras 7 e 8 ).

**Figura 7:** Paciente de 38 anos com COVID-19, apresentando febre, tosse seca, mal-estar e cefaleia há 6 dias. Tomografia computadorizada demonstrando sinal do halo invertido nos lobos inferiores

**Figura 8:** Paciente de 43 anos com COVID-19, apresentando falta de ar, febre e tosse há 8 dias. Tomografia computadorizada demonstrando sinal do halo invertido nos lobos inferiores

## CONSOLIDAÇÃO

A consolidação é a segunda alteração mais comumente encontrada na pneumonia causada pela COVID-19, após as opacidades em vidro fosco, e tende a predominar em fases mais tardias da infecção – sobretudo após o décimo dia.^( 10 - 12 )^Muitas vezes, entretanto, aparecem em conjunto. Ela representa o preenchimento dos alvéolos por exsudato inflamatório. Tal achado radiológico é caracterizado como aumento da densidade pulmonar com obscurecimento dos vasos e das linhas intersticiais em seu interior e possui formato frequentemente arredondado nessa pneumonia viral^( 14 , 15 )^( Figuras 9 a 11 ). O padrão evolutivo de vidro fosco, pavimentação em mosaico e consolidações é demonstrado na figura 12 , que também evidencia bandas parenquimatosas residuais no último controle, achados que vêm sendo descritos na fase tardia de convalescência desses pacientes.^( 11 )^

**Figura 9:** Paciente de 51 anos apresentando tosse seca e cefaleia, há 7 dias, além de febre, há 5 dias. Tomografia computadorizada mostrando opacidades em vidro fosco difusas, associadas a consolidações periféricas nos lobos pulmonares inferiores

**Figura 10:** Paciente de 41 anos com COVID-19 apresentando febre, tosse e dispneia há 5 dias. Tomografia computadorizada mostrando consolidações periféricas nos lobos superiores

**Figura 11:** Paciente de 28 anos com COVID-19 apresentando tosse há 15 dias. Tomografia computadorizada mostrando consolidação periférica no lobo inferior esquerdo

**Figura 12:** Tomografias de paciente de 44 anos com COVID-19. (A) Exame com 1 dia de doença, mostrando consolidações periféricas; (B) Exame com 5 dias de doença, mostrando significativo aumento da extensão e da densidade das opacidades pulmonares, predominando o aspecto de consolidação, ocupando grande parte dos lobos inferiores; (C) Exame com 10 dias de doença, mostrando redução da extensão e da densidade das opacidades pulmonares, com redução significativa do componente consolidação, persistindo bandas parenquimatosas pulmonares esparsas na periferia dos campos inferiores – achados habitualmente descritos na fase tardia da doença

## BRONCOGRAMA AÉREO

O broncograma aéreo é a identificação da luz brônquica aerada, no interior de uma consolidação alveolar.^( 14 , 15 )^Radiologicamente, é caracterizado como estrutura tubular hipoatenuante em meio ao parênquima pulmonar consolidado ( Figura 13 ).

**Figura 13:** Paciente com 51 anos com COVID-19 apresentando febre e tosse há 5 dias. Tomografia computadorizada mostrando opacidades em vidro fosco periféricas, consolidações posteriores com broncogramas aéreos de permeio

## LINFADENOMEGALIA

As linfadenomegalias torácicas são caracterizadas por linfonodos mediastinais e/ou hilares, medindo mais de 1cm em seu menor eixo axial. A TC só nos fornece as dimensões e a morfologia desses linfonodos, não sendo possível a diferenciação com processo neoplásico primário e/ou secundário. Linfonodomegalias não são comuns na COVID-19^( 14 , 15 )^( Figura 14 ).

**Figura 14:** Paciente com 52 anos com COVID-19 apresentando dispneia, febre e mialgia há 3 dias. Tomografia computadorizada demonstra linfonodomegalia hilar à direita (seta)

## CONCLUSÃO

Embora o diagnóstico de COVID-19 só possa ser confirmado por meio da reação em cadeia polimerase, a tomografia computadorizada pode auxiliar na avaliação da extensão da doença, das possíveis complicações e na determinação de diagnósticos alternativos. Para tanto, é importante que a equipe envolvida no atendimento conheça os achados sugestivos de pneumonia viral compatíveis com COVID-19.
